# Osteoarthritis In Vitro Models: Applications and Implications in Development of Intra-Articular Drug Delivery Systems

**DOI:** 10.3390/pharmaceutics13010060

**Published:** 2021-01-05

**Authors:** Carlota Salgado, Olivier Jordan, Eric Allémann

**Affiliations:** 1 School of Pharmaceutical Sciences, University of Geneva, 1211 Geneva, Switzerland; carlota.salgado@unige.ch (C.S.); olivier.jordan@unige.ch (O.J.); 2 Institute of Pharmaceutical Sciences of Western Switzerland, University of Geneva, 1211 Geneva, Switzerland

**Keywords:** osteoarthritis, intra-articular drug delivery systems, synovium, cartilage, in vitro cellular models, synoviocytes, chondrocytes

## Abstract

Osteoarthritis (OA) is a complex multi-target disease with an unmet medical need for the development of therapies that slow and potentially revert disease progression. Intra-articular (IA) delivery has seen a surge in osteoarthritis research in recent years. As local administration of molecules, this represents a way to circumvent systemic drug delivery struggles. When developing intra-articular formulations, the main goals are a sustained and controlled release of therapeutic drug doses, taking into account carrier choice, drug molecule, and articular joint tissue target. Therefore, the selection of models is critical when developing local administration formulation in terms of accurate outcome assessment, target and off-target effects and relevant translation to in vivo. The current review highlights the applications of OA in vitro models in the development of IA formulation by means of exploring their advantages and disadvantages. In vitro models are essential in studies of OA molecular pathways, understanding drug and target interactions, assessing cytotoxicity of carriers and drug molecules, and predicting in vivo behaviors. However, further understanding of molecular and tissue-specific intricacies of cellular models for 2D and 3D needs improvement to accurately portray in vivo conditions.

## 1. Introduction

Osteoarthritis (OA) is a chronic disease with worldwide incidence in the population aged 65 years and higher, representing a significant economic burden in terms of global health [1,2]. As the most common form of arthritis and one of the leading causes of disability in the elderly population, OA is characterized by chronic inflammation, articular cartilage degeneration and structural changes of whole joints. There is currently an unmet need for disease-modifying drugs (DMOADs) that slow or even revert disease progression [3,4,5]. Pharmacological treatment options focus on symptom management. Oral analgesic and nonsteroidal anti-inflammatory drugs (NSAIDs) are first-line treatments for pain and inflammation. However, since OA mainly affects the joint as a whole closed structure, systemic drugs result in less than optimal efficacy rates [6,7]. A known alternative that circumvents most of the drawbacks associated with systemic drug administration is the delivery of drugs locally, by intra-articular (IA) injection. IA allows for higher drug doses and prolonged delivery of drug molecules directly into affected joints. By this approach, more effective relief of symptoms may be attained, while systemic adverse effects are generally avoided. Different drug delivery systems (DDSs) have grown in the field to improve the delivery of small molecules locally to joints. These include different formulations such as polymeric nano and microparticles, hydrogels, liposomes and micelles, which have been extensively reviewed [8,9,10,11]. Due to its local administration, maintaining the selectivity of drug molecules and the carrier system towards biological tissue targets in the joint while avoiding off-target effects is critical when developing IA formulations. In this regard, the design of predictive in vitro OA models is crucial in characterizing and understanding the studied drug delivery systems for OA treatment. Different cellular models represent different tissues of the joint: synoviocytes, the synovium, and chondrocytes are used to model articular cartilage [12]. The different types of in vitro cellular models (i.e., monolayer, three-dimensional or explant) have various applications according to the final goals of IA formulation. Thus, a deep understanding of their intricacies is very important in this field. The purpose of the present review is to discuss the relevance of the different in vitro OA models in the development of IA formulations for OA treatment. At first, an overview of the latest (5 years) intra-articular DDSs is presented, highlighting the choice of in vitro model for each formulation. In this review, viscosupplementation formulations and delivery of cells (mesenchymal stem cells and platelet rich plasma) have been excluded. The review focuses on nano and micro carriers, hydrogels and liposomes containing drug molecules. Next, advantages and disadvantages, as well as possible readable markers and targets of different in vitro OA models, are discussed, based on their relevance for the development of intra-articular formulations.

## 2. Osteoarthritis

**Figure 1:**
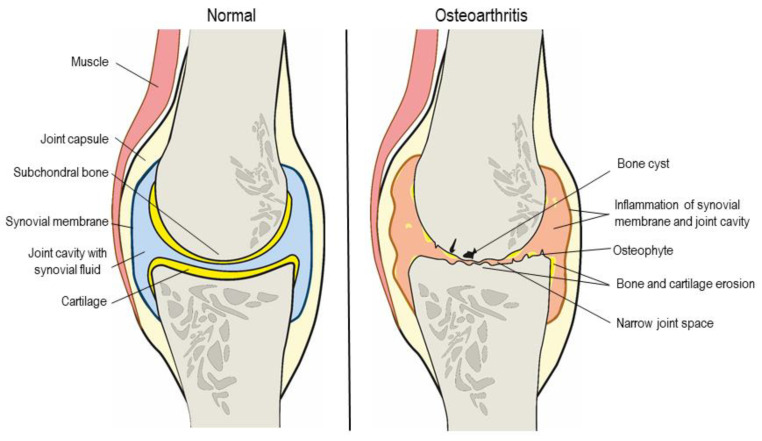
Schematic representation of the structural composition of a healthy and an osteoarthritic knee.

Osteoarthritis is a chronic degenerative disease of the whole joint. It is characterized by chronic inflammation, articular cartilage degeneration and structural changes in several joint tissues. Age >65 years old, obesity, gender (double prevalence in females), previous joint injuries and genetic predisposition to joint complications are all considered risk factors in the development of mild to severe OA [2,13]. Other than the economic burden it represents, OA is one of the primary causes of disability in the elderly population. Considered the most common form of arthritis, its worldwide incidence has repercussions on more than 100 million people [1,14]. The etiology of OA is unknown (primary OA) in the majority of cases, with secondary OA (one that follows joint injury) as an example of how trauma to the joint influences further disease progression. Several biomechanical and molecular processes are known to kick-start the pathology cycle. Tissue alterations of articular cartilage from increased cell proliferation and microarchitectural changes to the structure of subchondral bone are considered key events [15]. In early stages, degradation products of proteoglycan and collagen are released into the joint cavity from hyaline cartilage. This phenomenon stimulates immune cells from the synovial membrane to release pro-inflammatory cytokines—mainly IL-1β, IL-6 and TNFα. This inflammatory state induces catabolic mechanisms by the chondrocytes that produce matrix metalloproteinase (MMPs) 1, 3 and 13 and aggrecanases 1 and 2 (disintegrin and metalloproteinase with thrombospondin motifs—ADAMTS). Cartilage is further degraded, and the inflammatory state is perpetuated. Due to its poor vascularization and low cellular density, cartilage has a limited regeneration turnover. As disease advances, catabolic mechanisms outweigh those of repair by the extracellular matrix (ECM) [16,17,18]. As a result, there is a narrowing of the joint space due to cartilage degradation, subchondral bone erosion with the formation of osteophytes and small cysts, inflammation of the synovium (synovitis), and overall joint function loss (Figure 1). Clinical manifestations of the disease with well-established symptoms, mainly joint pain, stiffness and, consequently, a decrease in daily movement, appear relatively late. When detected and adequately diagnosed by physical assessment and bioimaging (X-ray, MRI), OA has often progressed to a stage where preventive and possibly reverting measures are no longer efficacious, leaving symptom management as the only option [19]. Currently, there is a substantial unmet need for disease-modifying OA drugs (DMOADs) that actively slow disease progression as no molecule of the sort has been approved or introduced in the market. Throughout the management of OA, different non-pharmacological treatments are adopted, like physical therapy, weight management and the use of different dietary supplements. Pharmacological treatment regimens depend on disease stage (I to IV, minimal to severe). Analgesics like paracetamol and nonsteroidal anti-inflammatory drugs (NSAIDs) such as diclofenac are first-line treatments. However, the drawbacks and adverse effects associated with the use of these systemic drugs are limiting [20,21,22]. In further stages of the disease, local administration (intra-articular) is an alternative that circumvents these issues. This local administration of hyaluronic acid derivatives, known as viscosupplementation, combines pain relief and improvement of joint motion from the greater cushioning effect provided by the hydrogels. Other biological compounds, like injections of autologous platelet-rich plasma have also been explored as local treatment of OA [23,24]. When symptom management is no longer viable in later stages, full joint replacement surgery of hip, knee or heel is an option [5,6].

## 3. Intra-Articular Drug Delivery Systems and Interactions with OA Joints

In a clinical setting, despite progress in OA research and development of disease-modifying drugs, joint anatomy and physiology still pose a challenge for effective drug delivery. Systemic drug delivery is challenging due to the poor irrigation and limited permeability of the synovial membrane and articular capsule of affected joints. Local, intra-articular administration of small molecules and larger protein products directly as solutions into joints is hindered by low retention times due to fast clearance [25]. Therefore, IA administration is not used to deliver common analgesic and anti-inflammatory drugs to the joint. As approved and in use, IA is only applied to deliver glucocorticoids (GC) and hyaluronic acid (HA) for viscosupplementation [26,27,28]. The lack of broader use of IA administration as a drug delivery route for OA treatment might be due to some drawbacks such as formulation issues and the invasiveness of the procedure, which limit the number of yearly injections. Thus, attaining drug loadings high enough to release sufficient therapeutic drug doses over extended periods represents a critical challenge [29]. Drug delivery systems with extended-release properties help circumvent these issues and others, like potential low aqueous solubility of many molecules. Comprehensive reviews on formulation aspects of IA DDSs have been published in recent years [26,28,30,31]. Table 1 shows the drug delivery systems investigated in the past five years to treat OA by IA administration. Different types of formulations—micro- and nanoparticles, hydrogels, liposomes—allow for controlled and extended release of drug and increased retention times in joints while avoiding systemic side effects [10,32,33]. Various classes of molecules have been investigated as DMOADs or for symptom management: analgesic/anti-inflammatory, chondroprotective/regenerative and bone resorption inhibitors [28]. Each category is linked to different target tissues in the joint. For example, anti-inflammatory drugs like celecoxib target the synovium, and chondroprotective drugs like kartogenin target articular cartilage tissue [34,35]. It is essential to consider tissue specificity when formulating IA drug delivery systems as off-target effects may occur and negatively impact OA progression. Understanding the structure of the different tissues of joints is thus key for the design of DDSs. Human joints are complex structures that connect bones, allowing the body’s movement. The main structures of synovial joints (diarthrosis, joints with movements) (Figure 1) are joint capsule, synovial membrane, joint cavity with synovial fluid, cartilage, ligaments, muscles, bursae, tendons, subchondral bone, nerves and vessels [36,37]. Synovial joints are the most affected by OA, with two of the main features that make them unique being key explored targets of therapeutic treatments: the synovial membrane and the articular cartilage. Synovium or synovial membrane is the connective tissue that lines the joint cavity. This heterogeneous tissue mainly comprises two types of synoviocytes: type A macrophage-like synoviocytes, lesser in number and increased in inflammatory conditions, have an important role in phagocytosis and production of pro-inflammatory cytokines; and type B fibroblast-like synoviocytes, the structural cells of the synovium (75% of cellular total), producing synovial fluid and ECM components. Collagen fibers, fenestrated blood capillaries and lymph vessels are other structures found in the inner layers of the membrane. The synovial fluid, produced by ultrafiltration of plasma, nourishes the non-irrigated articular cartilage, lubricates and absorbs shock [36,38]. Drug delivery systems with drugs targeting the synovium, like TSG-6 (TNFα gene precursor) or VX-745 (p38 MAPK inhibitor) are active on type B synoviocytes and macrophages, mostly through inflammatory and pain pathways [39,40]. On the other hand, articular cartilage (hyaline cartilage) is a connective tissue layer that lines the ends of the bones of the joint, serving as a barrier to friction and shock between them. Contrary to the synovium, this is an avascular, alymphatic and aneural tissue. It is composed of chondrocytes (differentiated mature cells) and ECM, mainly collagen and elastin fibers, aggrecan and proteoglycans. Its form and elasticity are determined by the organization of the collagen fibers, proteoglycans and diffusion of water molecules during movement. The lubricants and hyaluronic acid secreted both by the synovial fluid and chondrocytes are shock-absorbing and provide a cushioning effect. This cross-talk between tissues and synovial fluid is driven by mechanical load caused by body movement. Cartilage is part of the osteochondral unit as it covers the sub-chondral bone plate [41,42]. Examples of drugs targeting cartilage and bone delivered by IA administration of delivery systems include kartogenin (a chondrogenesis inductor from the RUNX-1 pathway) and doxycycline (an antibiotic with MMP inhibitor functions) [43,44].

The successful development of an IA drug delivery system formulation greatly depends on its interaction with the target tissue in the joint. Accurate choice and design of in vitro models of OA are crucial in understanding target interaction, predicting in vivo outcomes, and developing effective IA formulations.

## 4. In Vitro Models of OA

Grasping the complexity of OA pathophysiological mechanisms remains a challenge in OA research and negatively reflects on the successful development of DMOADs. Many OA in vitro and in vivo models have been developed and refined over the years. However, there is still no confirmed gold standard in vitro model to apply when developing OA drug molecules and/or drug delivery systems [12]. The establishment of accurate in vitro models is crucial as these influence choice of in vivo OA models. Although there are relevant in vivo OA animal models, major gaps in translation from animal to human OA conditions still prevail. Smart design and choice of in vitro models could potentially help bridge these gaps by enhancing predictability of OA models. Current OA in vivo models have additional limitations. These models often actively portray either post-traumatic and/or late-stage (III/IV) OA, leaving a large gap in understanding the spontaneous occurring disease and its early stages, where slowing disease progression would be an attractive treatment strategy. Sustainability and 3R initiatives (refinement, reduction and replacement) have to be considered to assess the usefulness of these in vivo animal models, where in vitro OA models can become the best alternative [86]. In this case, result translation and predictability from in vitro to in vivo models still lack refinement and accuracy. The processes of naturally occurring OA in certain animal species have been proven similar to those of humans. Therefore, tissue collection from affected animals is essential in the development of in vitro and ex vivo early-stage OA models. Tissue collection in humans (articular cartilage or synovium) is complex due to several ethical and regulatory issues, and retrieval at early stages of the pathology is nearly impossible. Recovery of samples is restricted to patients undergoing total joint replacement surgeries where OA is far evolved [87,88]. In this context, various in vitro OA cellular models have been designed and explored: monolayer (2D), 3D with or without scaffolds and tissue explants. Each model is adapted to a unique target tissue of the joint and yields quantification of different markers (e.g., inflammatory cytokines, collagen type II, aggrecan or MMPs). In addition, each model has its intricacies with relevant advantages and disadvantages, discussed in Table 2. In the field of IA DDSs, these are important, especially in characterizing release mechanisms and cytotoxicity of carrier systems. In Table 2, the different applications of each model in IA DDS pre-clinical development are further described.

### 4.1. 2D Cellular Models

Two-dimensional cellular models can be described as monolayer culture (Section 4.1.1), when a single cell line is cultured or co-culture (Section 4.1.2) when two or more cell lines are cultured together in a monolayer.

#### 4.1.1. Monolayer Culture

Culturing cells in monolayer is a well-established, cost-effective method to obtain relatively fast, reliable, and high-throughput results. As OA in vitro models, these can be immortal lines or harvested primary cells cultured adherent to plastic flat surfaces. Their source can vary from murine, bovine to human. Table 2 lists the use of the most common cell lines: RAW 264.7 macrophages, human primary synoviocytes and human articular chondrocytes. To evaluate IA delivery systems, 2D models with cells like synoviocytes or chondrocytes that respond to cytokine stimulation (typically IL-1β, mimicking the inflammatory catabolic environment of OA) are thus ideal for screening of either anti-inflammatory or chondroprotective molecules from DDSs by quantification of several inflammatory and cartilage degradation markers: IL-6, TNF-α, PGE_2_, COX-2, NO, iNOS, MMP-1, MMP-3, MMP-13, and ADAMTS-5. These monolayer models (especially human cell lines) are also useful and largely explored for cytotoxicity and proliferation testing in local administration cases since they correspond to the direct cellular target [40,53,62,64,84]. Additionally, different signaling pathways can be explored from these models, such as the inflammatory NF-κB pathway in human chondrocytes [94]. Human bone marrow mesenchymal stem cells (HBMSCs) have the ability to de-differentiate to mature articular chondrocytes; thus, these are also used to quantify chondrogenesis through sulfated glycosaminoglycans (GAG), abundant in articular cartilage ECM content, gene expression of collagen II and aggrecan, in addition to the cytotoxicity and screening of molecules [35,73]. However, problems arise from culturing primary articular chondrocytes, as the actual cartilage tissue would require a three-dimensional cell growth, interacting with the ECM, in contrast to a flat surface (Table 2). Therefore, after a small number of passages, which limits the number of experiments and length of studies, de-differentiation tends to occur as cells change in phenotype and morphology from an orthogonal shape to an elongated shape, resembling fibroblast-like chondrocytes. This phenotype is known to produce collagen type I fibers instead of the collagen II fibers consistent with articular cartilage, an issue when using this type of monolayer model to assess cartilage growth from collagen II and aggrecan quantification. This lack of tissue-mimicking properties prevents 2D in vitro models from accurately mimicking intercellular and cell-to-ECM relationships. Not only this, but weight-bearing and mechanical-loading experiments, crucial in the understanding of OA as a pathology, are not easily explored using these models [12].

Grasping the complexity of OA pathophysiological mechanisms remains a challenge in OA research and negatively reflects on the successful development of DMOADs. Many OA in vitro and in vivo models have been developed and refined over the years. However, there is still no confirmed gold standard in vitro model to apply when developing OA drug molecules and/or drug delivery systems [12].

#### 4.1.2. Co-Culture

Monolayer culture of different joint cell lines is an alternative to improve intercellular relationship studies. Differently from monolayers of a single cell line, in co-culture where chondrocytes are incubated together with synoviocytes and stimulated by pro-inflammatory cytokines, cross-talk between cells happens through intercellular calcium and paracrine signaling, maintaining homeostasis of articular chondrocytes. Evaluation of effects of anti-inflammatory or chondroprotective molecules in articular cartilage is then higher in accuracy by the co-culturing of both cell types due to the preservation of these intercellular signaling pathways [89]. Chondrocytes incubated with osteoblasts help maintain cellular physiology and phenotype through paracrine signaling. This is an useful model in investigating the effects of chondroprotection (slowing of cartilage degradation) in bone remodeling [95]. Mesenchymal stem cells are interesting in co-culture as pluripotency leads to specific de-differentiation, allowing for different cellular pathways to be analyzed together with articular chondrocytes from cellular secreted markers [96]. However, despite advantages gained by culturing different types of cells together in terms of tissue-like maintenance of homeostasis and phenotypes, this in vitro model is subject to some of the same drawbacks as monolayer culture, notably, culturing in a flat surface and lack of growth structure. In addition, maintaining different cellular environments at the same time is expensive.

### 4.2. 3D Cellular Models

Three-dimensional cellular models can be classified into models without scaffold (Section 4.2.1), where cells are grown in pellets, and models with scaffold (Section 4.2.2), where cellular growth happens in an external platform (biologic or synthetic polymer).

#### 4.2.1. 3D Cellular Models without Scaffold

Three-dimensional cellular pellets circumvent some of the disadvantages of monolayer cultures, especially as they allow a structure, maintaining cellular growth in all dimensions and synthesis of articular cartilage ECM. In this approach, chondrocytes can be centrifuged together in conical bottom wells or tubes or cultured under stirring using bioreactors. Inducing cell clustering forms cartilage tissue-like pellets, after a specific incubation time, with sizes up to 5 mm [97,98]. These pellets can mimic articular tissue as a whole, providing insights into cell-to-cell and cell-to-ECM relationships. Like in a monolayer culture, HBMSCs pellets can replace 3D chondrocyte pellets. As an in vitro model for IA DDSs development, 3D pellets have been applied in the evaluation of chondrogenesis and chondroprotective effects after IL-1β stimulation by GAG content quantification and gene expression of collagen II, aggrecan, and MMPs [35]. A primary reason as to why pellets are not a standard in vitro OA model is linked to difficulties in maintaining 3D cellular cultures in terms of cost and quantity. 3D articular dedifferentiated chondrocytes are not associated with high proliferation rates and derive from low monolayer passages restricting cellular amounts. Culture media is supplemented with a high amount of growth factors and chondrogenic stabilizers, representing higher costs compared to monolayer culture [99]. Additionally, pellets have short viability spans, where nutrients have difficulties in penetrating the pellet, inducing cell death at its core. As a model for IA DDSs, interaction of formulations with the tissue as a whole is essential in characterizing target specificity. The inability to fully penetrate the pellets poses a limitation to the use of this model in the IA setting [92]. Bypassing these shortcomings is, however, made possible by establishing this type of 3D cellular growth in external structures—scaffolds.

#### 4.2.2. 3D Cellular Models with Scaffold

Cells can be cultured directly into external scaffolds, gaining three-dimensional features. As an in vitro model for IA DDSs development, this alternative has great potential for targeted delivery. Not only does it provide structural support for 3D cellular growth by mimicking features of joint structure, making it a good model of loading and weight-bearing in OA, as the nature of the scaffold (biologic or synthetic) can play a role in cellular growth and maintenance. The most commonly used scaffolds are hydrogels due to their high water content and the extensive ability to tailor their mechanical and physicochemical properties. Biopolymers like agarose, chitosan, alginate and hyaluronic acid have been applied to grow chondrocytes, mimicking articular cartilage, and osteoblasts, aiming to model the osteochondral plate. Combining the growth of both these types of cells has also been explored, forming bilayer scaffolds, in an attempt to represent the whole articular joint [100]. As such, and after cytokine stimulation and exposure to therapeutic molecules, different cartilage markers can be assessed by different assays: GAG content (alcian blue assay), collagen II, aggrecan, MMPs (gene expression analysis) and even pro-inflammatory cytokines (enzyme-linked immunosorbent assay ELISA) [44,85]. Rheological measurements (elastic Young’s and G moduli) help investigate the mechanical properties of chondrocytes in hydrogels (agarose) [77]. Synthetic hydrogels and polymers can be applied as scaffolds, with advantages like mechanical features and support. 3D printing has been applied in this field with promising results in cartilage regeneration [101,102]. Compared to 2D models, scaffold-based 3D culture is expensive, difficult to maintain and hard to standardize, given the many options for scaffolds. Depending on their nature, problems may arise with how these influence results. For example, biopolymer-based hydrogels may themselves have a chondroprotective effect on cultured chondrocytes, skewing effects of tested drugs. The nature of the scaffold may also translate into differences between in vitro and in vivo models. For instance, hydrogels are rich in water, unlike subchondral bones of joints; thus, the growth of osteoblasts in such scaffolds is not an accurate representation of in vivo conditions [88].

### 4.3. Explants

Explants could be considered the most accurate in vitro model of OA as the whole tissue is maintained in its form and function. Just like tissue where cells are harvested from, their source can be both animal and human. Explants of both cartilage and synovial membrane are useful to investigate anti-inflammatory/chondroprotective effects of DDSs or molecules. Bovine cartilage is also commonly harvested to test the permeation and distribution of a drug or DDSs into the cartilage and/or subchondral bone, using fluorescent-dye-labeled-nanoparticles drug molecules. Femoral heads are attractive in loading and weight-bearing studies, whereas osteochondral plugs are used to investigate the balance between cartilage and bone regeneration when exposed to chondroprotective drugs. By measuring DNA and cellular turnover, cell viability and proliferation can also be assessed using explants after exposure to DDSs and/or free drug molecules [9,57]. Despite clear advantages (Table 2) from using explants where intercellular and cell-to-ECM relationships are preserved; extraction induces cell death on the outer layers of the tissue, compromising the model. Accurate induction of OA may pose another limitation, as often harvested tissues are healthy specimens and not pathological as the ones collected in other cellular models (monolayer, for example). Additionally, the maintenance of tissues in culture can be expensive and difficult to control, with explants lasting up to 10 days. Conditions such as temperature, pH, humidity, culture medium and supplements like insulin plus light exposure are crucial in maintaining the viability of explants. Another substantial limitation of this model is that viable replicates are very difficult to achieve, as tissue sources are finite and not abundant [87].

### 4.4. Considerations on OA In Vitro Models for Development of IA DDSs

Understanding the advantages and disadvantages of the different types of OA in vitro models is crucial when developing intra-articular drug delivery systems. The choice of in vitro model is influenced by how effects of the delivered drug can be assessed, be it anti-inflammatory by quantification of released cytokines or chondroprotection by evaluating GAG content and collagen II mRNA expression. Different cell lines such as macrophages, synoviocytes or chondrocytes secrete different factors and/or respond differently to cytokine stimulation. When evaluating the anti-inflammatory effects of therapeutic molecules in DDSs, macrophages and synoviocytes represent the most accurate cellular model. For chondroprotective effects and/or subchondral bone protection, it is important to test these in accurate representations of articular cartilage and subchondral bone. For this, 3D chondrocyte models or bilayer scaffolds for osteochondral defects are adequate models. Furthermore, articular cartilage or subchondral bone cells/tissue do not participate in inflammatory cascades directly, making these cellular models specific for measuring cartilage degradation markers. When developing, for example, an IA DDSs eluting a drug that has the synovium as a specific target, it is important to measure not only off-target activity in the other joint tissues but also the response of cartilage, for example, to the effects of the drug in the synovium. Monolayer models, though abundantly investigated and easy to establish, fail in the evaluation of cross-talk and molecular relationships within the different joint structures, especially important when developing a local delivery system. However, 2D models are relatively easy and accurate in assessing cytotoxicity and influence in cell proliferation of both drug and carriers. In contrast, 3D models allow for a more accurate and translational representation of joint tissues as phenotype and cellular growth are preserved. 3D models are essential in evaluating cytokine stimulation, cell-to-cell and cell-to-ECM relationships and, in the case of scaffold-based 3D models, loading studies, as these have mechanical properties not found in monolayer cultures. Nonetheless, their establishment requires highly specific expertise and can be costly. In addition, the accuracy and reproducibility of the outcomes, from cytotoxicity assays to gene expression analysis, can be high when applying 3D models. Explants from specific tissues are good representations of in vivo joints, as their intact features allow for loading and penetration studies of both IA carriers and free drug molecules. However, representative experimental replicates are not easily accessible, and molecular alterations may arise from the extraction of the tissues. As mentioned previously, time and duration play an important role in the development and application of in vitro models. OA is a slowly progressing, chronic disease where molecular changes often only result in actual physical symptoms very late. As such, tackling the effect over time on tissues is crucial to understand disease mechanisms and potential therapeutic options. However, experimentally, it is challenging to maintain cells and tissues viable for long periods of time. Bioreactors or tissue-mimicking polymers could help circumvent viability issues, maintaining OA conditions for slightly extended periods [103].

Formulation aspects also influence the choice of in vitro OA model. The formulation of DDSs (Table 1) implies the use of a carrier for a certain drug molecule. Carriers have an impact in terms of size and nature. In terms of size, the local administration of nano-range carriers (nanoparticles) can induce phagocytosis and inflammatory cascade from synoviocytes in the joint capsule [104]. Therefore, interactions at the cellular level when testing these DDSs are important to consider if macrophages/synoviocytes are the chosen in vitro cellular models. As previously discussed, most cellular OA models are cultured on plastic surfaces, in well plates, dishes or tubes. Micro-range carriers (microparticles or larger liposomes) are prone to sedimentation in these cell culture settings, especially polymeric carriers, which display high density when in a culture medium suspension. This sedimentation may negatively impact experimental result, by uneven drug molecule distribution, heterogeneous presentation to test cells and lower contact surfaces between the carrier–drug complex and cells [105]. This issue can be bypassed by performing experiments in orbital shakers. However, as described for 3D cellular models, altering centrifuge force and balance induces changes in cellular growth and phenotype [100]. When evaluating hydrogels (Table 1), either in monolayer or 3D cellular models, even when using explants, it is important to consider the nature of the polymer (synthetic or bio) and the viscosity of the gel. Like for nano-/microcarriers, choice of polymer will have an impact on cellular response. Thus, biocompatibility and innocuousness of polymers are important characteristics, particularly when testing inflammation and anti-inflammatory effects, as further induction of inflammatory cascades is undesired. The majority of hydrogels being explored for OA treatment are HA-based, a natural component of articular cartilage [10,69,70,72,73,75,78]. As such, it is important to assess their impact as stand-alone carrier vs. carrier with drug, as it is expected that this type of gel will have an influence on chondrocyte growth by inducing chondroprotection through CD44 receptor interaction. Lastly, rheological properties of hydrogels need to be considered when applying in vitro cellular models. High viscosity may induce occlusion effects in either cultured cells or tissues, generating hypoxia phenomena and thus lowering viability scores [106]. Consideration of all different formulation aspects does not exclude testing of drug-alone controls in these cellular OA models, as these dictate why and how DDSs are better alternatives in IA administration.

## 5. Conclusions and Future Perspectives

Improved design and development of efficacious IA DDSs relies on the use of accurate, predictive in vitro and in vivo models. However, to date, there is no OA gold standard in vitro model and few guidelines or models adapted specifically to IA DDSs formulations. Presently, monolayer models, despite being easy to establish and ideal for rapid screening of molecules, fail in representing accurate OA conditions, such as cross-talk between different tissues. This could be bypassed by co-culture of two types of cell lines, like synoviocytes and chondrocytes, but aspects like cell de-differentiation and ECM growth are not negligible. Three-dimensional models are considered better representations of in vivo OA, as in these models, three-dimensional structures of tissues and cellular phenotype and growth are preserved. However, with or without scaffold, 3D models are difficult to establish and maintain, and outcomes vary greatly according to the source and nature of scaffold. For studies in articular tissues, explants are considered best in correlation to in vivo OA conditions. However, viable replicates and maintenance of tissues in in vitro environments are important limitations. Recently, a bioengineering approach combining 3D cell culture and microfluidics—organ-on-chip (OoC)—has been in the field of OA. Cartilage-on-chip and osteochondral-tissue-on-chip have been developed to perfectly mimic joint microenvironments, allowing for better reproductions of in vivo conditions. Promising results have been described testing the drug alone, making this a promising approach for the better development of IA DDSs in the future [107,108,109]. In this context, considerations (Table 2) have to be taken into account when designing and developing IA DDSs, especially when deciding outcome readouts. To this extent, the type of formulation and mode of action of drug molecules (Table 1) play a critical role. Monolayer models are better suited for testing anti-inflammatory activity, whereas 3D chondrocyte models are preferred to evaluate chondroprotection activities. When testing hydrogels, it is important to assess the nature of the scaffold in 3D models and even occlusion in explants. In the future, research advancements should focus on improving the design and development of OA in vitro models for better prediction of in vivo and, eventually, clinical results. This should be done while always considering the tailoring of in vitro models to specific IA DDSs formulations, like maintaining cellular viability conditions for testing of sustained prolonged drug release delivery systems.

## Figures and Tables

**Table 1:** Intra-articular small molecule drug delivery systems for OA treatment, developed in the past 5 years (Acronyms defined at the bottom of the table).

Formulation	Drug	Carrier	Type of Study	Main Target Tissue	In Vitro Model	In Vivo Model	Authors; Year; References
**Microparticles**	Doxycycline	PCL	Pre-clinical studies	Cartilage	3D rabbit chondrocyte agarose model	Rabbits	Aydin et al. 2015 [44]
	Celecoxib	PEA	Pre-clinical studies	Synovium	Differentiated HI-60 cells and lysates	Human synovium and synovial fluid (ex vivo); rat ACLT model	Janssen et al. 2016 [32]
	Etoricoxib	PCL	Pre-clinical studies	Synovium, cartilage	Not reported	Rats	Arunkumar et al. 2016 [45]
	Lornoxicam	Chitosan/TPP	Pre-clinical studies	Synovium, cartilage	Not reported	Rat MIA model	Abd-Allah et al. 2016 [46]
	Fluvastatin	PLGA	Pre-clinical studies	Cartilage	Human primary chondrocytes	Rabbit ACLT model	Goto et al. 2017 [47]
	Rhein (cassic acid)	PLGA	Pre-clinical studies	Synovium	THP-1 macrophages	Not reported	Gomez-Gaete et al. 2017 [8]
	Kartogenin	PLA	Pre-clinical studies	Cartilage	Human synoviocytes	Mice DMM model	Maudens et al. 2018 [43]
	PH-797804, Dexamethasone	PLA	Pre-clinical studies	Synovium	Human synoviocytes	Mice AIA model	Maudens et al. 2018 [48]
	Triamcinolone acetonide (Zilretta™)	PLGA	Phase II/III clinical trials in OA patients ^1^	Synovium, cartilage	Not reported	Rat knee model ^2^	Kumar et al. 2015 ^2^; Kraus et al. 2018 ^1^ [49,50]
	TSG-6 (tumor necrosis factor-alpha stimulated gene-6)	Heparin	Pre-clinical studies	Cartilage	Not reported	Rat MMT model	Tellier et al. 2018 [39]
	Fluticasone propionate	PVA	Pre-clinical studies	Synovium	Not reported	Beagle dogs	Getgood et al. 2019 [51]
	Celecoxib	PLA	Pre-clinical studies	Synovium	Human synoviocytes	Not reported	Salgado et al. 2020 [52]
	Rapamycin	PLGA	Pre-clinical studies	Cartilage	Human immortal chondrocytes	Mice	Dhanabalan et al. 2020 [53]
**Nanoparticles**	VX-745 (p38 MAPK inhibitor)	PLA and PLGA	Pre-clinical studies	Synovium	Human synoviocytes	Mice AIA model	Pradal et al. 2015 [40]
	Dexamethasone	Avidin/PEG	Pre-clinical studies	Synovium, cartilage	Bovine knee cartilage explants	Not reported	Bajpayee et al. 2016 [54]
	KAFAK (anti-inflammatory mitogen-activated protein kinase-activated protein kinase 2 (MK2)-inhibiting cell-penetrating peptide)	pNiPAM-PEG	Pre-clinical studies	Synovium, cartilage	Bovine knee cartilage explants	Not reported	Lin et al. 2016 [9]
	Kartogenin; Diclofenac	Chitosan/Pluronic F127	Pre-clinical studies	Synovium, cartilage	Human BMSCs (bone marrow mesenchymal stem cells); Human primary chondrocytes	Rats	Kang et al. 2016 [35]
	Curcumin	PLGA	Pre-clinical studies	Synovium, cartilage	Not reported	Rats	Niazvand et al. 2017 [55]
	Dexamethasone	Avidin	Pre-clinical studies	Synovium	Not reported	Rabbit ACLT model	Bajpayee et al. 2017 [56]
	KAFAK	pNiPAM-PEG	Pre-clinical studies	Synovium, cartilage	RAW 264.7 macrophages; Bovine knee cartilage explants	Not reported	McMasters et al. 2017 [57]
	CAP (chondrocyte affinity peptide)	PEG-PAMAM	Pre-clinical studies	Cartilage	Human primary chondrocytes	Rats	Hu et al. 2018 [58]
	Kartogenin	Polyurethane	Pre-clinical studies	Cartilage	Rat primary chondrocytes	Rat ACLT model	Fan et al. 2018 [59]
	Adenosine	PEG-b-PLA	Pre-clinical studies	Synovium, cartilage	RAW 264.7 macrophages	Rat ACLT model	Liu et al. 2019 [60]
	Etoricoxib	PLGA-PEG-PLGA	Pre-clinical studies	Synovium, cartilage	Human primary chondrocytes	Rat ACLT model	Liu et al. 2019 [33]
	Hyaluronic acid	PLGA	Pre-clinical studies	Cartilage	RAW 264.7 macrophages	Brine shrimp; Rats	Mota et al. 2019 [61]
	Hyaluronic acid and near-infrared dye	PLGA	Pre-clinical studies	Cartilage	Human primary chondrocytes	Mice DMM model	Zerrillo et al. 2019 [62]
	Celastrol	Mesoporous silica	Pre-clinical studies	Cartilage	Rat primary chondrocytes	Rat MIA model	Jin et al. 2020 [63]
	Diacerein	PLGA	Pre-clinical studies	Synovium, cartilage	Rat synoviocytes	Rat MIA model	Jung et al. 2020 [64]
	Etoricoxib	PLA/Chitosan	Pre-clinical studies	Synovium	MC3T3-E1 cells (mouse osteoblast precursor)	Not reported	Salama et al. 2020 [65]
	MK2i (anti-inflammatory MK2-inhibiting peptide)	Linked and non-linked NIPAm	Pre-clinical studies	Synovium, cartilage	Bovine primary chondrocytes	Rats	Deloney et al. 2020 [66]
	Oxaceprol	PLGA	Pre-clinical studies	Synovium	Human primary LCLs (lymphoblastoid cell lines)	Not reported	Alarçin et al. 2020 [67]
	Triamcinolone acetonide	Dextran sulfate conjugated	Pre-clinical studies	Synovium	RAW 264.7 macrophages; L929 cells (mouse fibroblast)	Mice MIA model	She et al. 2020 [68]
**Hydrogels**	Amphotericin B	Hyaluronic acid/glyceryl monooleate	Pre-clinical studies	Synovium, cartilage	Not reported	Rabbits	Shan-Bin et al. 2015 [69]
	Celecoxib	PCLA-PEG-PCLA	Pre-clinical studies	Synovium	Not reported	Horse	Petit et al. 2015 [34]
	Methotrexate/dexamethasone/near-infrared dye	Hyaluronic acid + PLGA microcapsules	Pre-clinical studies	Synovium	RAW 264.7 macrophages	Rat RA model	Son et al. 2015 [70]
	Sinomenine hydrochloride	Phytantriol	Formulation studies	Not reported	Not reported	Not reported	Chen et al. 2015 [71]
	Dexamethasone	Hyaluronic acid	Pre-clinical studies	Synovium, cartilage	Human primary chondrocytes	Rat ACLT model	Zhang et al. 2016 [72]
	PEGylated Kartogenin	Hyaluronic acid	Pre-clinical studies	Cartilage	Human BMSCs; human primary chondrocytes	Rat ACLT model	Kang et al. 2017 [73]
	Celecoxib	PCLA-PEG-PCLA	Pre-clinical studies	Synovium	Not reported	Equine synovitis model	Cokeleare et al. 2018 [74]
	Dexamethasone	Hyaluronic acid/pNiPAM	Pre-clinical studies	Synovium	Human synoviocytes	Mice DMM model	Maudens et al. 2018 [10]
	Triamcinolone hexacetonide (Cingal^®^)	Hyaluronic acid	Phase II/III clinical trials in OA patients	Synovium, cartilage	Not reported	Not reported	Hangody et al. 2018 [75]
	Simvastatin	Gelatin	Pre-clinical studies	Cartilage	Mouse primary chondrocytes	Mice	Tanaka et al. 2019 [76]
	Dexamethasone	Agarose gel + PLGA microspheres	Pre-clinical studies	Synovium, cartilage	3D canine articular chondrocyte construct	Canine osteochondral autograft model	Stefani et al. 2020 [77]
	Diclofenac	Hyalomer (HA and poloxamer 407)	Pre-clinical studies	Synovium, cartilage	Not reported	Rat MIA model	Hanafy et al. 2020 [78]
	Diclofenac	Linked PAPE (2-Pyridylamino substituted 1-phenylethanol)	Formulation studies	Not reported	Not reported	Not reported	Kawanami et al. 2020 [79]
	Eicosapentanoic acid	Gelatin	Pre-clinical studies	Synovium	Human primary chondrocytes	Mouse DMM model	Tsubosaka et al. 2020 [80]
	Hyaluronic acid/diclofenac sodium	Silica colloidal crystal beads- pNiPAM	Pre-clinical studies	Synovium, cartilage	Human primary chondrocytes	Rat DMM model	Yang et al. 2020 [81]
**Liposomes**	Quercetin	mPEG-PA (Methoxy-poly(ethylene glycol)-l-poly(alanine))	Pre-clinical studies	Synovium, cartilage	Human primary chondrocytes	Rat ACLT model	Mok et al. 2020 [82]
	Fish oil protein encapsulated in gold nanoparticles	DPPC	Pre-clinical studies	Synovium	Not reported	Rats	Sarkar et al. 2019 [83]
	Glucosamine sulphate	Distearoyl phosphocholine	Pre-clinical studies	Cartilage	Mouse primary chondrocytes	Not reported	Ji et al. 2019 [84]
	Rapamycin	DSPC combined with low-intensity pulsed ultrasound	Pre-clinical studies	Cartilage	Human primary chondrocytes	Guinea pigs	Chen et al. 2020 [85]

Acronyms: PCL: polycaprolactone; PEA: polyetheramine; PLGA: poly(lactic-co-glycolic acid); PLA: polylactic acid; PVAL: poly(vinyl alcohol); PCLA: poly(ε-caprolactone-co-lactide); PEG: polyethylene glycol; pNiPAM: poly(N-isopropylacrylamide); PAMAM: poly(amidoamine); DPPC: dipalmitoylphosphatidylcholine; DSPC: 1,2-distearoyl-sn-glycero-3-phosphocholine; MIA: monoiodoacetate; AIA: antigen-induced arthritis; ACLT: anterior cruciate ligament transection; DMM: destabilization of medial meniscus.

**Table 2:** Overview of advantages and disadvantages of in vitro OA models and their application in IA DDSs development.

In Vitro OA Model		Advantages	Disadvantages	Models Applied in IA DDS Development (as per Table 1)	Outcome Evaluation (as per Table 1)
**2D cellular culture**	**Monolayer**	High throughput, low cost. Homogenous cell exposition to nutrients. Allows for differences in cellular phenotype studies [12]	Furthest from natural in vivo tissue conditions. High variability (different passages). Better suited for synoviocytes than chondrocytes. 2D substrate induces de-differentiation and changes in morphology [12]	- Synoviocytes (human, mouse and rat) - Chondrocytes (human, murine, rat and bovine) - Macrophages (human and murine)BMSCs (human) - BMSCs (human)	RAW 264.7 macrophages [33,57,60,68,70]: - Cytotoxicity assays - Quantification of NO. cAMP, IL-6, IL-1β and TNF-α Synoviocytes [10,40,43,48,52,64]: - Cytotoxicity and proliferation - Quantification of IL-6, PGE_2_, IL-1β, TNF-α, MMP-3, MMP-13, COX-2 and ADAMTS-5 Chondrocytes [47,53,59,62,63,66,72,73,81,84]: - Cytotoxicity, apoptosis and proliferation assays - Quantification of IL-6, IL-1β, TNF-α, GAG/DNA, Aggrecan, Collagen II, MMP-1, MMP-3, TAC-1, MMP-13, COX-2, PGE_2_, iNOS and ADAMTS-5 - Senescence assays after genotoxic and oxidative stress [53] - Expression of inflammatory transcription factors: p-IKKα/β [80]
	**Co-culture**	Important in studies of cell-to-cell interactions and studies of influence of different cellular phenotypes together [12]	Expensive and difficult to maintain. Lacks in three-dimensional characteristics of cartilage growth [87]	(examples not included in Table 1) - Synoviocytes-chondrocytes - Chondrocytes-osteoblasts [89,90]	
**3D cellular culture**	**Without Scaffold**	High similarity with in vivo tissue conditions as it maintains structure from ECM growth. Cellular phenotype is preserved. Important in studies of intercellular and cell to ECM relationship and loading capacity assays [88,91]	Expensive and difficult to maintain. Restricted throughput (hard to propagate without compromising cell quality). Nature of scaffold plays role in cellular growth [92]	- Chondrocyte pellets - Hanging drop BMSCs	- Quantification of: GAG/DNA, Collagen II, Aggrecan [35]
	**With Scaffold**			- Hydrogels: biomaterial and synthetic - Polymeric scaffolds (osteochondral plugs) - Micro- and nanocarriers - Fiber/Mesh scaffolds [88]	- GAG/DNA, MMP-13 and hydroxyproline quantification; proliferation in agarose assay by DNA quantification [44] - GAG/DNA, Collagen II and Young’s/dynamic modulus (Eγ and G) [77] - Proliferation in alginate beads - Quantification of IL-6, MMP-13, Collagen II and Aggrecan [85]
**Explants**		Easy to obtain and inexpensive. Allows for studies of intercellular and cell to ECM relationship because it maintains tissue as a whole [93]	High variability and limited amounts of replicates from source. Cell death at edge of extracted tissues [12]	- Articular cartilage and synovial membrane (human and bovine) - Osteochondral plugs (human) - Femoral chondyles (human, murine and equine)	- Cytotoxicity and cartilage penetration assays - Quantification of IL-6 [9,57]

## Data Availability

Not applicable.
